# The effects of high-intensity interval training/moderate-intensity continuous training on the inhibition of fat accumulation in rats fed a high-fat diet during training and detraining

**DOI:** 10.1186/s12944-024-02209-7

**Published:** 2024-07-22

**Authors:** Yu Liu, Lukai Zhang, Qiqi Wang, Hui Liu, Xiangui Zhu, Hong Li, Haifeng Zhang

**Affiliations:** 1 Physical Education College, Hebei Normal University, Shijiazhuang, China; 2 Hebei Provincial Key Lab of Measurement and Evaluation in Human Movement and Bio- Information, Hebei Normal University, Shijiazhuang, China

**Keywords:** HIIT, MICT, Detraining, Adipose tissue

## Abstract

**Background:**

Compared with moderate-intensity continuous training (MICT), high-intensity interval training (HIIT) has at least a comparable effect on inhibiting an increase in fat. However, few studies have been conducted to examine the effects of detraining on body fat in rats fed a high-fat diet. The present study aimed to compare the effects of 10 weeks of HIIT or MICT as well as 6 weeks of detraining on body fat in rats fed a high-fat diet.

**Methods:**

After being fed a high-fat diet for 8 weeks, 54 female rats were randomly assigned to six groups: (1) CON-10, sedentary control for 10 weeks; (2) MICT-10, 10 weeks of MICT; (3) HIIT-10, 10 weeks of HIIT; (4) CON-16, sedentary control for 16 weeks; (5) MICT-16, 10 weeks of MICT followed by 6 weeks of training cessation; and (6) HIIT-16, 10 weeks of HIIT followed by 6 weeks of training cessation. The training was performed 5 days/week. The subcutaneous adipose tissue (inguinal; SCAT), visceral adipose tissue (periuterine; VAT) and serum lipid profile were analysed after 10 or 16 weeks. Adipose tissue triglyceride lipase (ATGL) protein expression in VAT was assessed by western blotting.

**Results:**

HIIT-10 and MICT-10 prevented the increase in SCAT, VAT and serum lipid levels seen in the CON group. During the 6-week detraining period, HIIT continued to prevent the increase in adipose tissue mass observed in the CON group, whereas MICT at least maintained this inhibition. The inhibition of fat mass increase was mainly the result of preventing adipocyte hypertrophy. The HIIT-10 and HIIT-16 groups showed the highest ATGL protein expression.

**Conclusions:**

HIIT has a comparable effect to MICT on inhibiting fat accumulation in female rats; however, the inhibition of SCAT and VAT increase by HIIT is superior to MICT after short-term training cessation.

**Graphical Abstract:**

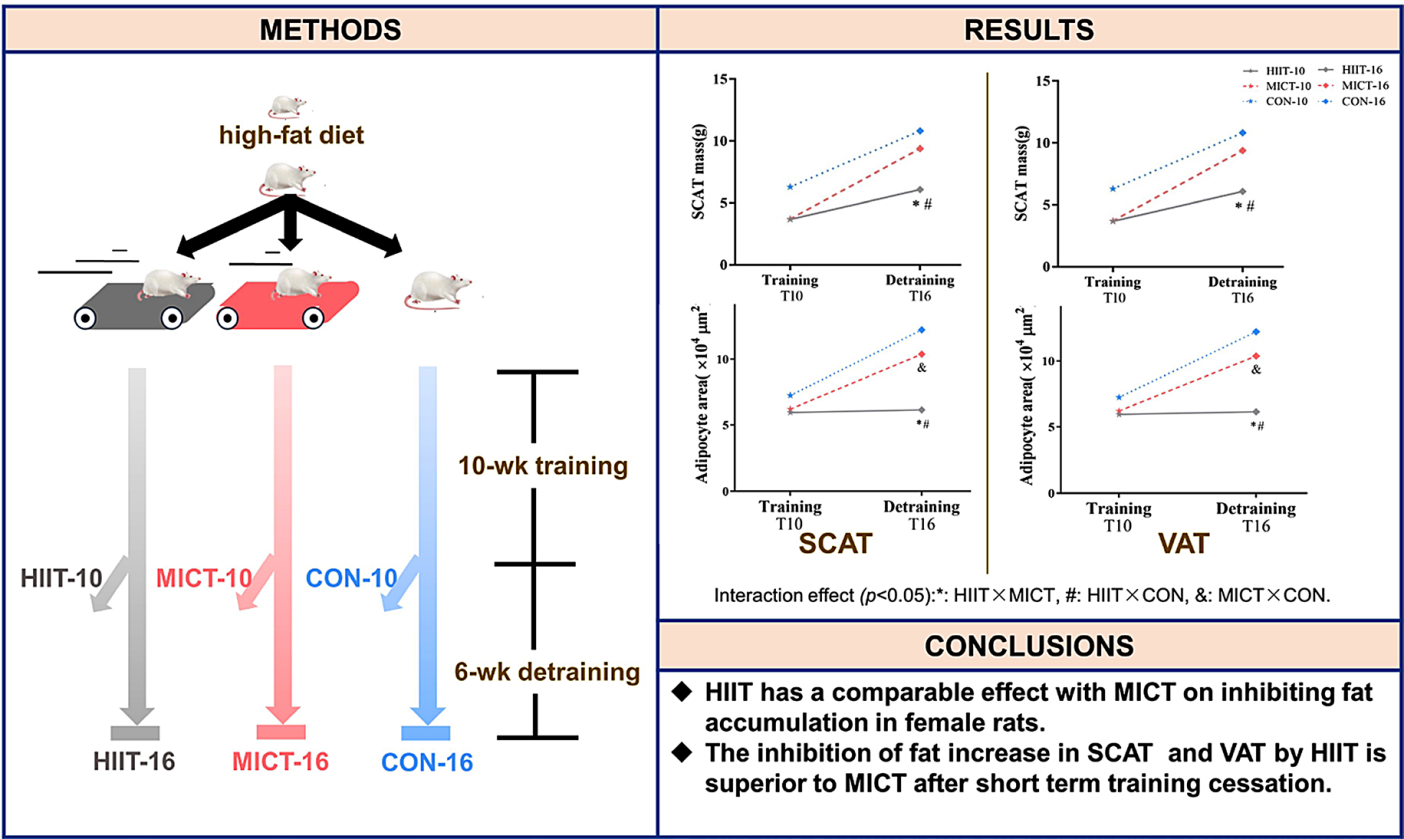

**Supplementary Information:**

The online version contains supplementary material available at 10.1186/s12944-024-02209-7.

## Background

Obesity/overweight is closely related to several chronic diseases, including type 2 diabetes, fatty liver and cardiovascular diseases [1, 2]. Moderate-intensity continuous training (MICT), which predominantly utilises fat as the main metabolic substrate, is a non-pharmacological way to control weight [3]. However, training cessation may lead to a fat rebound and thus influence the choice of training modality [4]. A recent meta-analysis reported that there was an increase in fat mass resulting from the enlarged adipocytes after MICT cessation [5]. Based on the compensatory effect, fat consumed during the training period may be re-synthesised when people with obesity stop training [6, 7]. Moreover, MICT significantly enhanced the activity of adipose triglyceride lipase (ATGL) after 2 weeks of detraining [8, 9].

Recent studies have shown that high-intensity interval training (HIIT) – which has a lower time commitment – exerts fat loss effects that are at least comparable to MICT, especially regarding the visceral adipose tissue (VAT) [10]. However, individuals may pause training, resulting in partial or total loss of the metabolic and physiological benefits they gained from the training [11, 12]. It is unknown whether the physiological adaptions on fat reduction are different after interruption of HIIT and MICT. Given that HIIT reduces fat more effectively than MICT [10, 13], HIIT seems to outperform MICT in combating fat accumulation after training cessation. HIIT utilises more glycogen and less fat than MICT; however, most studies have demonstrated that HIIT enhances excess post-exercise oxygen consumption (EPOC) and the resting metabolic rate, resulting in greater fat burning in individuals with obesity or overweight [14, 15]. The substantial amount of glycogen consumed during HIIT needs to be replaced after exercise by burning more fat through the liver–adipose tissue axis. A large amount of glycogen consumed by skeletal muscle is supplemented by hepatic gluconeogenesis after acute HIIT. Enhanced hepatic gluconeogenesis involves increased lipolysis of adipose tissue and release of glycerol [16, 17]. Moreover, HIIT increases lipase activity in VAT more than MICT [18].

Previous studies have indicated that HIIT provides better maintenance in Maximum Oxygen Uptake (VO_2max)_ and a more robust anorectic in individuals with overweight after detraining [10]. It is possible that there are also beneficial effects in adipose tissue. It is hypothesised that long-term HIIT leads to a lower risk of fat rebound during the detraining period. The present study assigned rats fed a high-fat diet to HIIT or MICT followed by short-term detraining to compare their maintenance of fat loss. The findings were expected to provide insights into physiological adaptations after training cessation and offer guidance on training modalities for sustained weight management in women with obesity who discontinue training for a period of time.

## Methods

### Study design

**Fig. 1:**
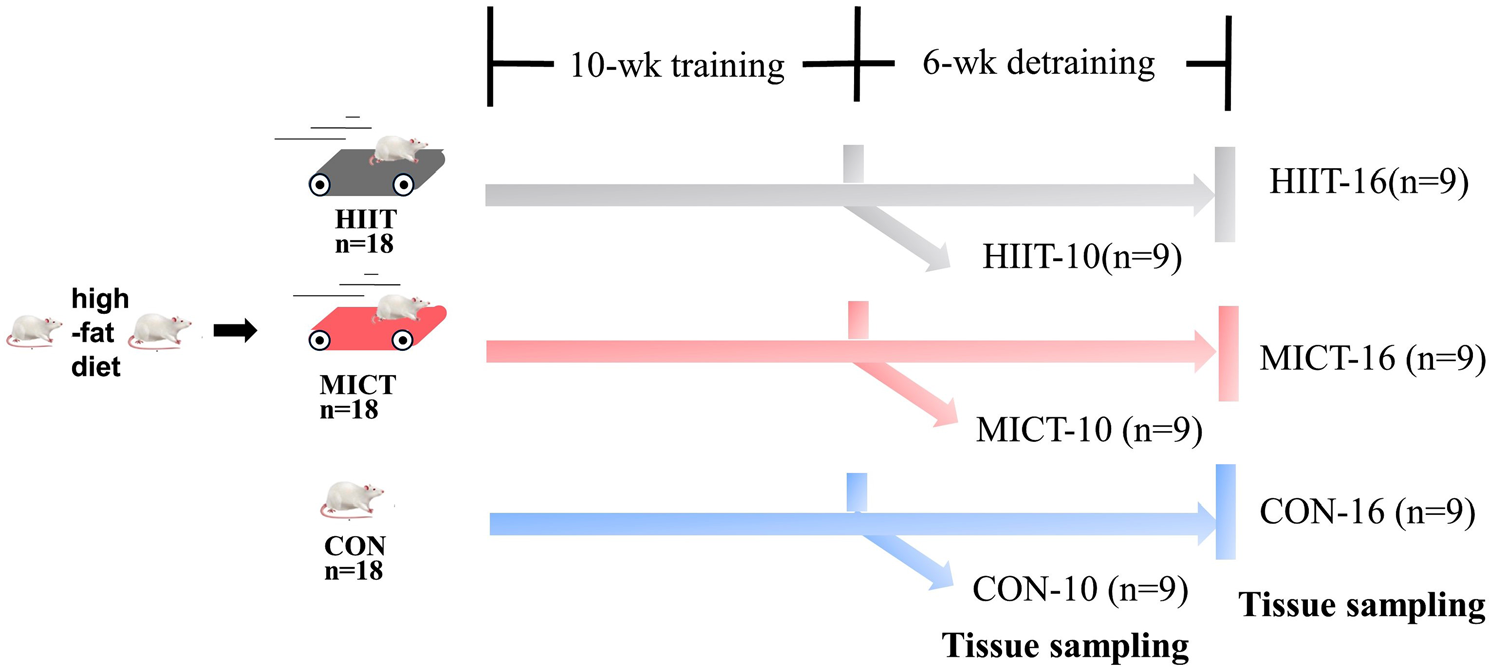
Experimental design. Abbreviations: CON-10, sedentary control for 10 weeks; MICT-10, 10 weeks of moderate-intensity continuous training (MICT); HIIT-10, 10 weeks of high-intensity interval training (HIIT); CON-16, sedentary control for 16 weeks; MICT-16, 10 weeks of MICT followed by 6 weeks of training cessation; HIIT-16, 10 weeks of HIIT followed by 6 weeks of training cessation

Fifty-four female Sprague Dawley rats (7 weeks old and 174.24 ± 7.95 g) were housed singly at 22 ± 2℃, 50% ± 5% relative humidity and a 12-h photoperiod. They were fed a high-fat diet (60% chow diet, 18% protein powder, 16% sugar, 5% fat and 1% sodium cholate [19–21]; see Fig. S1 for detailed information on the diet) for 8 weeks. Then, they were randomly divided into the following groups: (1) CON-10 (*n* = 9), sedentary control for 10 weeks; (2) MICT-10 (*n* = 9), 10 weeks of MICT; (3) HIIT-10 (*n* = 9), 10 weeks of HIIT; (4) CON-16 (*n* = 9), sedentary control for 16 weeks; (5) MICT-16 (*n* = 9), 10 weeks of MICT followed by 6 weeks of training cessation; and (6) HIIT-16 (*n* = 9), 10 weeks of HIIT followed by 6 weeks of training cessation (Fig. 1). The rats continued to be fed the high-fat diet throughout the study. The body weight, the food intake and voluntary running wheels were measured at the each end of week.

### Exercise protocol

After 7 days of adaptive training (10 min/day, 5 m/min), each rat in the HIIT-10, MICT-10, HIIT-16 and MICT-16 group was subjected to an incremental exercise test (IET) to determine the maximum running speed (S_max_). The rats were subjected to a 15° uphill treadmill at the initial speed of 9 m/min, after which it increased by 2 m/min for 2 min until exhaustion (inability to complete the test despite physical poking). The speed at this stage was defined as S_max_ [22]. The high- and moderate-intensity running speed was 85–90% S_max_ and 50–70% S_max_, respectively. Each MICT session lasted 45 min. Each HIIT training session comprised alternating 1-min intervals of running at the high-intensity speed and 2-min intervals of running at the moderate-intensity speed. The HIIT session duration was adjusted so that the HIIT and MICT sessions included an equal running distance. All training was performed 5 days/week. After the last session of weeks 2, 4, 6 and 8, the IET was repeated to adjust the running intensity. During the detraining period, the rats were confined to their cages, but were allowed to engage voluntarily with a wheel.

### Sample collection

Forty-eight hours after the last training session and after fasting overnight, the rats were anaesthetised (intraperitoneal pentobarbital sodium, Solarbio, China) and then euthanised at the end of 10th (T10) and 16th week (T16). Approximately 10 ml of blood was drawn from the apex of heart and transferred to a centrifuge tube. The blood samples were allowed to clot for 30 min and then centrifuged at 3500 rpm for 20 min. The serum was extracted from the supernatant of the processed blood samples and stored at -80 °C until analysis. The subcutaneous adipose tissue (inguinal; SCAT) and visceral adipose tissue (periuterine; VAT) were excised and weighed. Half of the aforementioned fat pads were fixed in 4% paraformaldehyde for haematoxylin and eosin (H&E) staining. The rest of the VAT was frozen at -80 °C for western blotting.

### Histological assessment of adipose tissue

The number of adipocytes and their size (area) were assessed after H&E staining. In brief, adipose tissue was embedded in paraffin after fixation. Then, the embedded tissue was sectioned at 6–8 μm, dewaxed, rehydrated, stained with haematoxylin, washed with water, subjected to alcohol dehydration and finally stained with eosin. Following dehydration and sealing, the prepared microscope slides were examined under an optical microscope. Each group consisted of nine samples; three fields with clear and intact cell membranes were chosen for each sample. The distance between each visual field was at least 200 μm. Image J 1.8.0 (National Institutes of Health, USA) was used to measure and analyse the number and diameter of adipocytes.

### Serum lipid profile

Blood samples were analysed for total cholesterol (TC), triglycerides (TG), low-density lipoprotein cholesterol (LDL-C) and high-density lipoprotein cholesterol (HDL-C) using assay kits (Jiancheng Bio, China).

### Western blotting for ATGL in VAT

Cryopreserved VAT was homogenised with radioimmunoprecipitation assay (RIPA) buffer (Solaribo) containing protease and phosphatase inhibitors. Then, the homogenate was centrifuged (4℃, 12,000 rpm, 20 min) and the supernatant was removed. The protein concentration was determined with the bicinchoninic acid assay. Ten micrograms of total protein was combined with loading buffer, incubated at 98℃ for 5 min and then subjected to sodium dodecyl sulphate–polyacrylamide gel electrophoresis with a 4–10% gel. The resolved protein was transferred onto the polyvinylidene difluoride membrane (Millipore). The membrane was incubated with a primary antibody against ATGL (Abcam, England) or GAPDH (as a loading control). Then, it was incubated with a secondary antibody (Servicebio, China). Fusion FX were used to analyse the blot. Image J 1.8.0 was used to analyse the grey value.

### Statistical analyses

The data are expressed as the mean ± standard deviation. Repeated-measures analysis of variance (ANOVA) was used to analyse the changes in body weight and food intake during the training and detraining period. The food intake, voluntary running wheels, the mass of adipose tissue, the adipocyte numbers and areas, the grey value of oil red O staining and the protein expression at the end of training and detraining were compared using the a 3 (groups: CON, MICT and HIIT) × 2 (time: 10 weeks [T10] and 16 weeks 9T16]) two-way ANOVA. If the main or interaction effect was significantly different, then the least significant difference (LSD) method was used for pairwise comparisons. *P* < 0.05 was considered statistically significant difference.

## Results

### Body weight, food intake and the number of voluntary wheel rotations

**Fig. 2:**
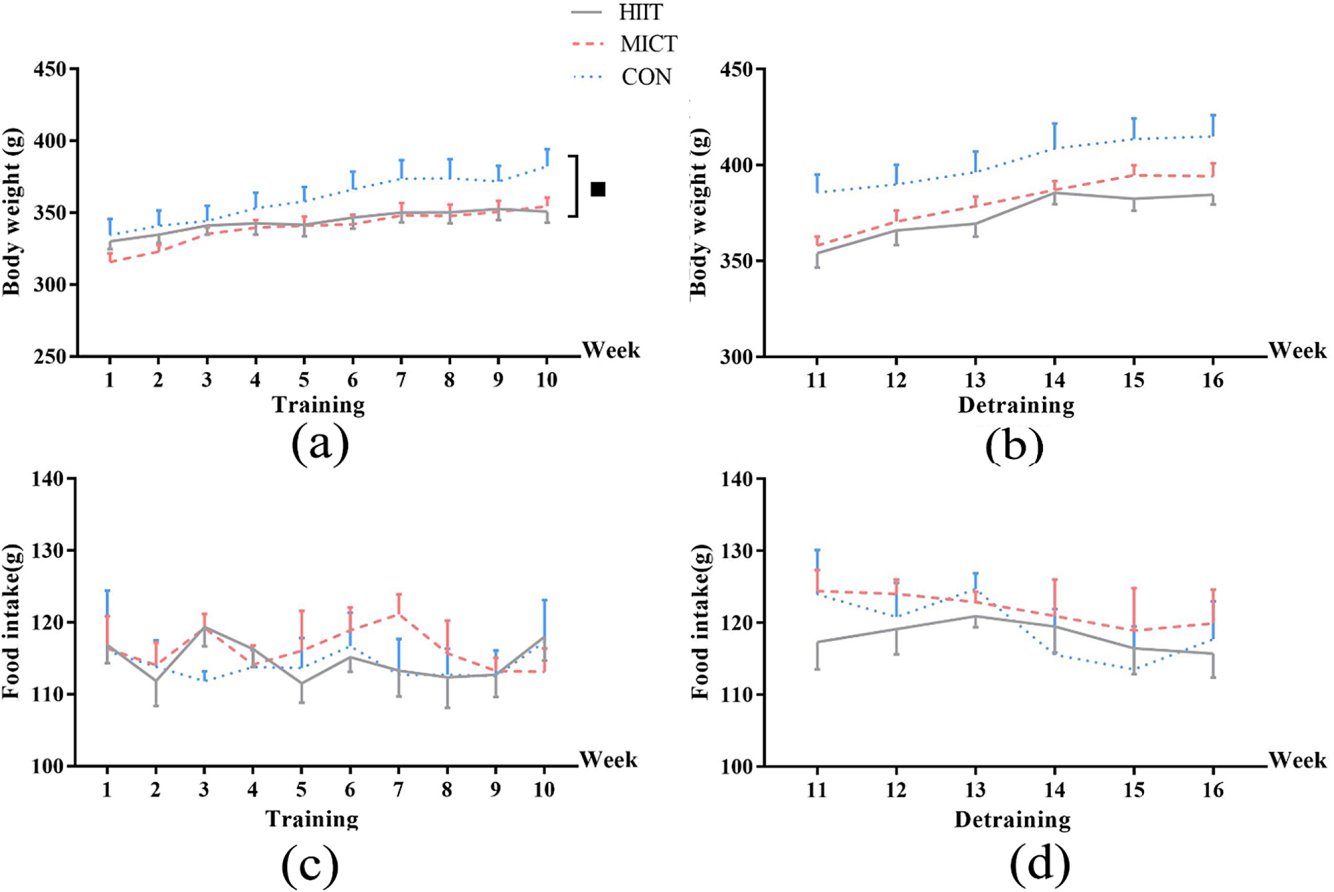
The average body during the (**a**) 10-week training and (**b**) 6-week detraining periods. The average food intake during the (**c**) 10-week training and (**d**) 6-week detraining periods. Abbreviations: CON, sedentary control; HIIT, high-intensity interval training; MICT, moderate continuous training. ■: time × group (*P* < 0.05)

Figure 2a and b shows the changes in body weight during the 10-week training and 6-week detraining periods, respectively. The average body was similar among all groups after the rats had been fed a high-fat diet for 8 weeks. After 10 weeks of training, the body weight in all three groups was significantly higher (*P* < 0.05) compared with baseline. Both training groups showed suppressed increase in body weight compared to the controls. At week 10, the average body weight was 354.39 ± 18.79 g for MICT-10 and 350.74 ± 23.07 g for HIIT-10, 8% and 9% lower, respectively, compared with the CON-10 group (382.30 ± 35.34; *P* < 0.05). At week 16, the HIIT-16 and MICT-16 groups still showed a significantly lower body weight compared with the CON-16 group maintained the effect of fat reduction (time × group interaction, *P* > 0.05). Table 1; Fig. 2c and d illustrate that the average daily food intake and number of voluntary wheel rotations did not show significant time, group or time × group interaction effects (*P* > 0.05).

**Table 1:** Food intake and the number of voluntary wheel rotations at weeks 10 (T10) and 16 (T16)

	Training period (T1–T10)			Detraining period (T10–T16)			Two-way analysis of variance					
							Time main effect		Group main effect		Interaction	
	CON-10 (*n* = 9)	MICT-10 (*n* = 9)	HIIT-10 (*n* = 9)	CON-16 (*n* = 9)	MICT-16 (*n* = 9)	HIIT-16 (*n* = 9)	F	*P*	F	*P*	F	*P*
Food intake (g/week)	114.91 ± 7.73	116.61 ± 11.63	114.46 ± 5.47	119.73 ± 16.77	121.90 ± 15.04	118.71 ± 10.55	0.244	0.784	2.182	0.146	0.008	0.992
The number of voluntary wheel rotation per day	2161.39 ± 596.76	1955.72 ± 606.55	2173.33 ± 738.48	1761.11 ± 502.33	1859.56 ± 311.05	2101.56 ± 273.71	1.077	0.356	3.075	0.092	0.957	0.398

Abbreviations CON-10, sedentary control for 10 weeks; MICT-10, 10 weeks of moderate-intensity continuous training (MICT); HIIT-10, 10 weeks of high-intensity interval training (HIIT); CON-16, sedentary control for 16 weeks; MICT-16, 10 weeks of MICT followed by 6 weeks of training cessation; HIIT-16, 10 weeks of HIIT followed by 6 weeks of training cessation

### SCAT and VAT mass

**Fig. 3:**
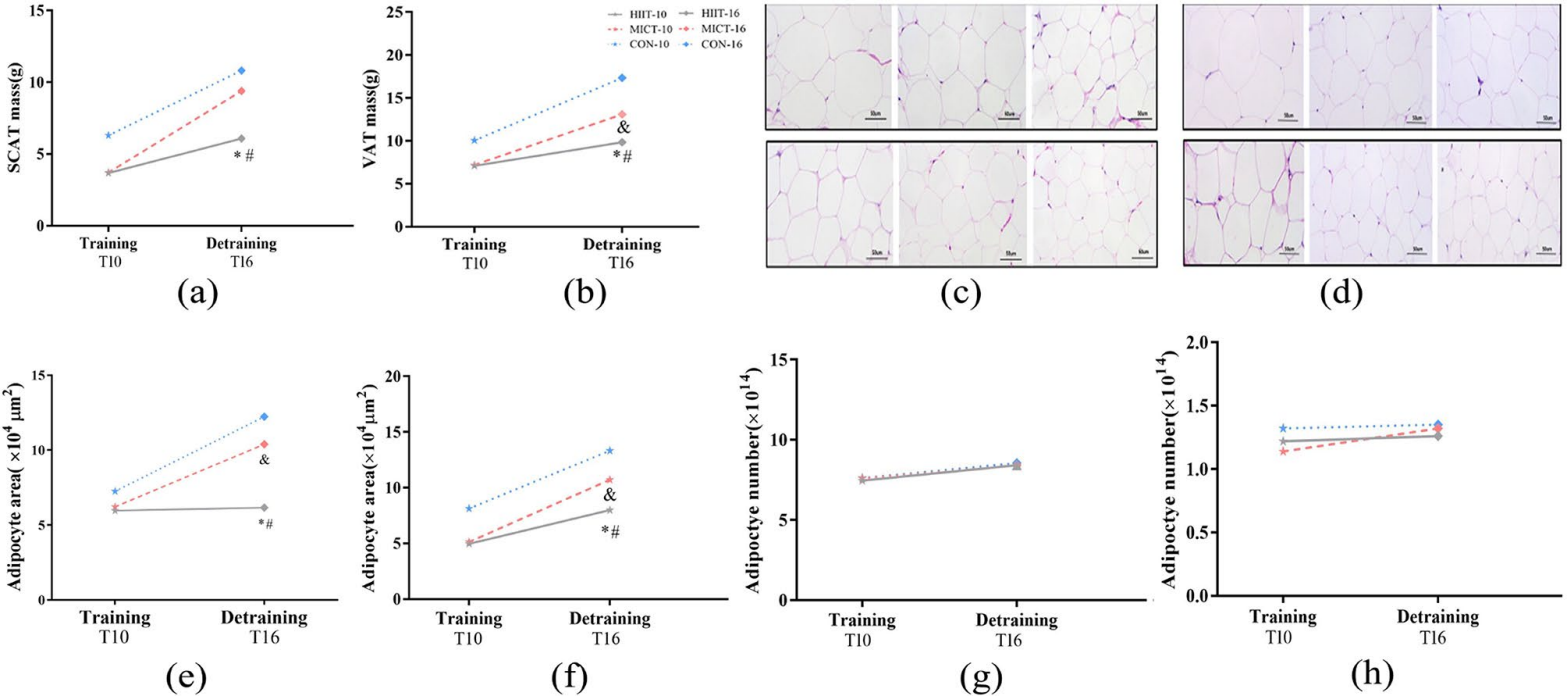
Changes in (**a**) subcutaneous adipose tissue (SCAT) mass and (**b**) visceral adipose tissue (VAT) mass. Representative images of adipocyte from (**c**) SCAT and (**d**) VAT. The images are magnified 400×, and the scale bar represents 50 μm. The adipocyte area in (**e**) SCAT and (**f**) VAT. The number of adipocytes in (**g**) SCAT and (**h**) VAT. Abbreviations: CON-10, sedentary control for 10 weeks; MICT-10, 10 weeks of moderate-intensity continuous training (MICT); HIIT-10, 10 weeks of high-intensity interval training (HIIT); CON-16, sedentary control for 16 weeks; MICT-16, 10 weeks of MICT followed by 6 weeks of training cessation; HIIT-16, 10 weeks of HIIT followed by 6 weeks of training cessation. * HIIT × time vs. MICT × time, *P* < 0.05; # HIIT × time vs. CON × time, *P* < 0.05; & MICT × time vs. CON × time, *P* < 0.05

The SCAT mass was significantly lower in the HIIT-10 (3.67 ± 0.95 g) and MICT-10 (3.71 ± 1.13 g) groups compared with the CON-10 group (6.29 ± 2.10 g, *P* < 0.05). The VAT mass was also significantly lower in the HIIT-10 (7.10 ± 1.74 g) and MICT-10 (7.19 ± 2.1 g) groups compared with the CON-10 group (10.05 ± 2.62, *P* < 0.05). There was no significant difference between the HIIT-10 and MICT-10 groups at week 10. As shown in Fig. 3a and b, after 6 weeks of detraining, HIIT continued to suppress the increase in VAT and SCAT mass (HIIT × time vs. CON × time, *P* < 0.05). MICT also maintained the inhibition of fat accumulation in SCAT (MICT × time vs. CON × time, *P* > 0.05) and continued to inhibit the increase in VAT mass (MICT × time vs. CON × time, *P* < 0.05). Moreover, the SCAT and VAT mass increase was lower in the HIIT-16 group than the MICT-16 group (HIIT × time vs. MICT × time, *P* < 0.05).

### Comparison of the number of area of adipocytes in SCAT and VAT

In theory, an increase in fat mass can be explained by inhibition of adipocyte hypertrophy and/or proliferation [14]. Figure 3c–f shows that the SCAT adipocyte area (×10^4^ µm^2^) was significantly lower in the HIIT-10 (5.96 ± 1.16) and MICT-10 (6.22 ± 1.11) groups compared with the CON-10 group (7.25 ± 0.98, *P* < 0.05). The VAT adipocyte area was also lower in the HIIT-10 (6.16 ± 1.22) and MICT-10 (10.39 ± 1.88) groups compared with the CON-10 group (12.23 ± 1.93, *P* < 0.05). After detraining, HIIT and MICT continued to inhibit adipocyte hypertrophy (HIIT/MICT × time vs. CON× time, *P* < 0.05). HIIT inhibited adipocyte hypertrophy more effectively than MICT (HIIT× time vs. MICT× time, *P* < 0.05). Two-way ANOVA indicated that the main and interaction effects were not significant (*P* > 0.05) for the number of adipocytes (Fig. 3g, h).

### ATGL expression in VAT

**Fig. 4:**
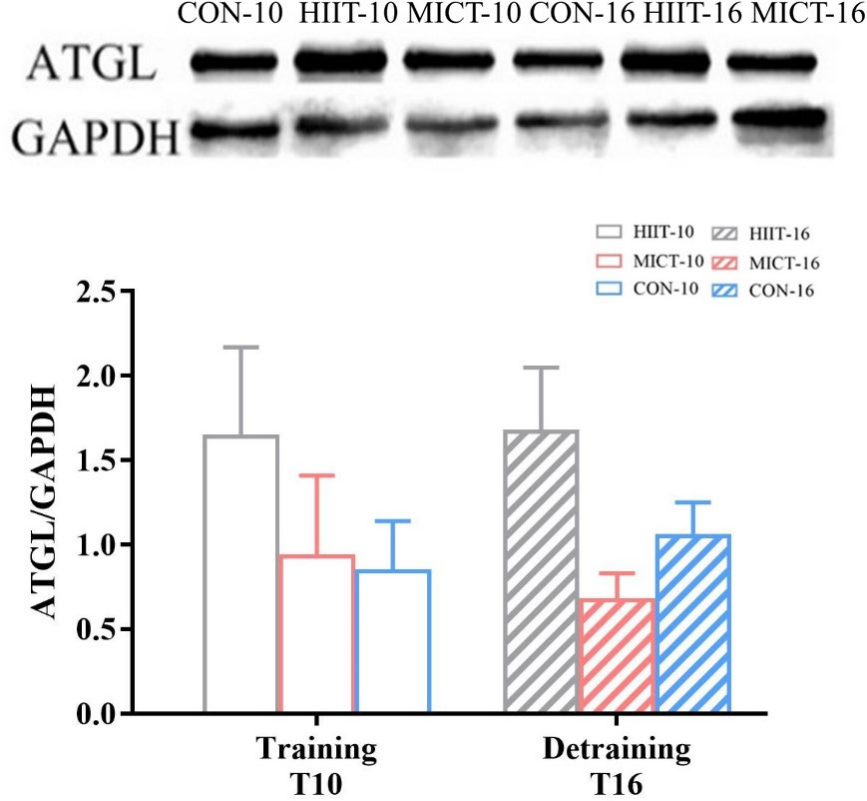
Adipose tissue triglyceride lipase (ATGL) expression in visceral adipose tissue. Abbreviations: CON-10, sedentary control for 10 weeks; MICT-10, 10 weeks of moderate-intensity continuous training (MICT); HIIT-10, 10 weeks of high-intensity interval training (HIIT); CON-16, sedentary control for 16 weeks; MICT-16, 10 weeks of MICT followed by 6 weeks of training cessation; HIIT-16, 10 weeks of HIIT followed by 6 weeks of training cessation

ATGL expression was significantly higher in the HIIT-16 group compared with the MICT-16 and CON-16 groups (*p* < 0.05; Fig. 4).

### Serum lipid profile

Serum TG and LDL-C were significantly lower in the HIIT-10 group compared with the CON-10 group (*P* < 0.05), and significantly lower in the HIIT-16 and MICT-16 groups compared with the CON-16 group (*P* < 0.05). There was no difference between the HIIT-16 and MICT-16 groups (*P* > 0.05). The main and interaction effects for TC and HDL-C were not significant (*P* > 0.05, Table 2).

**Table 2:** The serum lipid profile at weeks 10 (T10) and 16 (T16)

	The end of training (T10)			The end of detraining (T16)			Two-way ANOVA					
							Time effect		Group effect		Time × group interaction	
	CON-10 (*n* = 9)	MICT-10 (*n* = 9)	HIIT-10 (*n* = 9)	CON-16 (*n* = 9)	MICT-16 (*n* = 9)	HIIT-16 (*n* = 9)	F	*P*	F	*P*	F	*P*
TC (mmol/L)	2.25 ± 0.19	2.14 ± 0.30	2.13 ± 0.29	2.40 ± 0.40	2.39 ± 0.89	2.27 ± 0.50	0.259	0.773	1.523	0.224	0.074	0.929
TG (mmol/L)	0.89 ± 0.43	0.60 ± 0.05^*^	0.52 ± 0.17^*^	0.97 ± 0.42	0.72 ± 0.32^*^	0.60 ± 0.33^*^	5.946	0.005	1.106	0.299	0.014	0.986
LDL-C (mmol/L)	0.77 ± 0.22	0.51 ± 0.15^*^	0.41 ± 0.17^*^	0.74 ± 0.18	0.53 ± 0.27^*^	0.51 ± 0.11^*^	10.754	0.000	0.337	0.564	0.423	0.658
HDL-C (mmol/L)	2.22 ± 0.25	2.32 ± 0.29	2.33 ± 0.30	2.25 ± 0.35	2.25 ± 0.29	2.28 ± 0.41	0.197	0.822	0.071	0.791	0.102	0.904

Abbreviations: CON-10, sedentary control for 10 weeks; MICT-10, 10 weeks of moderate-intensity continuous training (MICT); HIIT-10, 10 weeks of high-intensity interval training (HIIT); CON-16, sedentary control for 16 weeks; MICT-16, 10 weeks of MICT followed by 6 weeks of training cessation; HIIT-16, 10 weeks of HIIT followed by 6 weeks of training cessation HDL-C, high-density lipoprotein cholesterol; LDL-C, low-density lipoprotein cholesterol; TC, total cholesterol; TG, triglycerides. * significant difference compared with the corresponding CON group (*P* < 0.05)

## Discussion

This study compared the effect of 10 weeks of HIIT or MICT with an equal running distance on fat reduction and further investigated their ability to maintain fat reduction after 6 weeks of detraining. The present results confirmed that HIIT and MICT had a comparable effect on inhibiting the increase in VAT and SCAT mass serum lipid levels (including TG and LDL-C) in rats fed a high-fat diet. However, HIIT exhibited a superior effect on inhibiting the increase in VAT and SCAT mass during training cessation, possibly due to the higher lipolytic activity.

### Effect of 10 weeks of training

Ten weeks of HIIT and MICT had a comparable effect on weight control in rats fed a high-fat diet, consistent with previous studies [23–25]. For individuals who are obese/overweight, HIIT is a more time-efficient strategy to reduce fat mass compared with MICT. This difference is due to higher lipolytic hormone levels (epinephrine, norepinephrine and growth hormone), increased fat oxidation and fibroblast growth factor 21 that could contribute to lipolysis in white adipose tissue after HIIT [26–29]. However, due to genetic polymorphisms, sensitivity to obesity varies despite consuming a high-fat diet. There may be inter-individual variability in fat loss caused by training and interruption of regular physical activity, which were not rule out in the present study [21, 30].

Central obesity is characterised by excess abdominal fat. VAT is a key risk factor for type 2 diabetes and cardiovascular disease [31]. In the present study, 10 weeks of HIIT or MICT significantly inhibited the increase in VAT mass. The primary source of TG in adipocytes is the intake of free fatty acids (FFA). Lipoprotein lipase (LPL) hydrolyses TG into FFA in the capillaries of skeletal muscle or adipose tissue; these FFA can then be transported into the cytoplasm [32]. ATGL is the main enzyme involved in the initial step of fat mobilisation: it hydrolyses TG into diglycerides and FFA. In the present study, only 10 weeks of HIIT upregulated ATGL expression compared. Consistently, the existing literature suggests that HIIT could be more effective in increasing ATGL activity in visceral white adipose tissue than MICT [8, 33]. However, the inhibition of fat accumulation might also be associated with the inhibition of lipogenesis [34]. The imbalance between apoptosis and adipogenesis, adipose tissue blood flow and the adipokines also influence fat accumulation [14]. Data in the present study did not clearly elucidate how 10 weeks of HIIT led to fat reduction. Previous research has confirmed that during the vigorous-intensity training, fatty acid mobilisation from adipose tissue may be suppressed. HIIT might act similarly and then stimulate greater oxidation of fat after exercise during post exercise period [35, 36]. Studies have shown that there is higher lipolytic activity 3 and 24 h after HIIT [17, 33].

The present study showed that chronic consumption of a high-fat diet increased body weight. Obesity/overweight occurs when the energy intake exceeds the energy expenditure [37]. Unlike MICT, long-term HIIT has been associated with reduced appetite and can maintain this effect after the suspension of training. However, neither training type affected the total food intake in other studies [38, 39]. Moreover, the present results showed that the weight control might not be attributed to the unchanged caloric intake and physical activity outside of exercise (Table 1).

### The effects of 6 weeks of detraining

Individuals who are obese/overweight might discontinue training due to time constraints or negative affective valence, potentially reversing the benefits of exercise [10, 40]. As the inactive duration is prolonged, it becomes increasingly challenging for rats fed a high-fat diet to overcome the metabolic adaptations achieved during the exercise period [7]. There have been a few studies examining the effect of HIIT compared with MICT after detraining, but there have been two studies focusing on the fat rebound [10, 39]. Ahmadizad et al. [39] and Gripp et al. [10] found that HIIT had respectively comparable or better maintenance in reducing the percent body fat and visceral fat than moderate continuous training in obese/overweight individuals after detraining. The present study differs from the above two studies. The present study showed that HIIT inhibited more fat increase than MICT in VAT and SCAT after detraining in rats, and further observed the changes of adipocytes and protein expression of relevant lipase.

In the present study, neither HIIT nor MICT altered food intake and voluntary physical activity during the 6-week detraining period. Ahmadizad et al. [39] showed that 6 weeks of moderate training and 1 week of detraining did not change voluntary activity. In another study, HIIT decreased appetite, but it did not change the total energy intake after detraining [39]. Therefore, the weight control in the present study cannot be explained by nutritional changes.

When performing moderate-intensity aerobic training, the mass of adipose tissue reduced by the exercise in adulthood is more associated with the inhibition of adipocyte hypertrophy rather than the inhibition of adipocyte hyperplasia [14, 41]. In the present study, the inhibition of SCAT and VAT mass increase after training and detraining was not due to inhibition of adipocyte proliferation. In other studies, however, the number of adipocytes returned to the pre-training level after detraining [14, 42]. Sertie et al. [34] found that a short detraining duration (4 weeks) after aerobic continuous training (1 h/day, 5 days/week, 8 weeks) increased the expression of peroxisome proliferators-activated receptors (PPARγ) and the number of adipocytes in rats fed a standard diet. It should be noted that a high-fat diet can also increase PPARγ expression [43]. Although the number of adipocytes increased after detraining, the change was not significant. Moreover, exhaustive exercise damages cellular DNA, which accelerates apoptosis and decreases the number of mature adipocytes, perhaps contributing to the reduction in adipose tissue mass and maintenance after training cessation [42, 44].

Regardless of whether a high-fat or standard diet is consumed, the adipocyte diameter following detraining indicates fat accretion [45]. In the present study, both HIIT and MICT inhibited the increase in adipose tissue mass by inhibiting the increase in adipocyte area after detraining. However, most studies have indicated that the inhibition of fat accumulation induced by the physical inactivity could disappear. Yasari et al. [46] found that obese adult rats that detrained for 6 weeks after 8 weeks of MICT showed increased intrabdominal fat deposition resulting from an increase in adipocyte diameter compared with sedentary rats. A shorter detraining duration (4 week) after aerobic continuous training (1 h/day, 5 days/week, 8 weeks) also induced hypertrophy in subcutaneous and visceral fat compared with sedentary control rats [34]. Sertie et al. [34] reported that the VAT mass rebounded after moderate-intensity exercise. They observed that the proportion of glucose oxidation in fat and fat synthesis in adipose tissue increased after detraining while lipolysis did not change significantly [34, 42, 47]. Agarwal et al. [48] also showed that discontinuing activity for 2 weeks after 6 weeks of MICT could not completely eliminate the beneficial effects of regular exercise and training cessation for a longer period may lead to a complete reversal of the beneficial effects.

HIIT might better maintain the beneficial effects obtained during training compared with MICT. Twelve weeks of MICT significantly reduced the levels of inflammatory factors such as interleukin 6 and tumor necrosis factor-α, increase antioxidant capacity and promoted fat metabolism in individuals with obesity. However, this inhibitory effect returned to the level of pre-exercise after 4 weeks of detraining [49]. HIIT may reduce inflammation more than MICT to better maintain fat reduction during detraining [49, 50]. Plasma nesfatin-1 (anorectic effect) levels during HIIT were higher and were maintained after 1-week training suspension, which may be related to the improvement of insulin sensitivity [39]. Moghadasi et al. also demonstrated that insulin resistance that inhibited lipolysis decreased after the suspension of high-intensity endurance training [51, 52]. High-intensity exercise could prevent the dramatic decline in resting metabolic rates after the training hiatus and would result in only modest rebound of body fat mass [53].

A previous study showed that sprint interval training with an intensity of > 100% V̇O_2max_ and HIIT reduced abdominal visceral fat mass more than MICT, accompanied by the release of serum growth hormone and epinephrine [54]. The inhibition of fat accumulation after HIIT is greater than after MICT due to the higher lipase activity associated with HIIT [28, 35, 55]. The activity of hormone-sensitive triglyceride lipase (HSL) was higher 12 h after HIIT compared with 12 h after MICT, which may be one of the reasons for increased fat burning and oxygen consumption associated with HIIT [17]. The present study found higher ATGL protein expression in VAT after detraining in the HIIT-16 group but not the MICT-16 group. Hence, the higher lipolytic activity induced by exercise might remain during the detraining period. Bae et al. [20] also showed that 6 weeks of moderate-intensity treadmill training reduced the visceral fat area in rats fed a high-fat diet compared with sedentary rats after 8 weeks of training suspension; this was accompanied by increased expression of ATGL and other lipid droplet–related signalling proteins, indicating increased lipolysis [56]. HIIT has been confirmed to have a superior influence on VAT reduction compared with MICT [26], perhaps due to preserved lipolytic activity.

In the present study, HIIT and MICT reduced serum TG and LDL-C after 10 weeks of training. After 6 weeks of detraining, the improvement in TG and LDL-C had been maintained. Consistently, researchers have found significant changes in serum lipid levels after training and detraining [57, 58]. HIIT leads to reduced lipoprotein lipase and ATGL expression after cessation of training, and therefore may reduce the LDL-C catabolism [58]. Similarly to the present study, Dinari et al. [59] found that the effect of MICT on improving LDL-C in diabetic rats was maintained after 4 weeks of detraining. The increased energy demand during exercise and the utilisation of TG as fuel was maintained after training cessation [58]. The present study found that neither HIIT nor MICT improved the TC and HDL-C. On the contrary, 12 weeks of HIIT (3 times/week, running 4 × 4 min at 90% maximal heart rate (HR_max_), interval 3 min at 65% HR_max_) increased HDL-C in middle-aged men with obesity compared with nonlinear resistance training; there was no rebound after 4 weeks of detraining. This HIIT protocol also had a better lipid-lowering effect [60]. HDL-C is one of the most sensitive parameters to aerobic exercise. Most studies have shown that HIIT is better at improving HDL-C compared with MICT after training cessation [58, 61, 62]. Kodama et al. [63] showed that there was a significant increase in serum HDL-C when the exercise duration was > 120 min or the external work done was at least 900 kcal per week. Therefore, the low volume of exercise may be the reason why HIIT and MICT had no effect on HDL in the present study.

### Strengths and limitations

The strength of the present study is the comparison of the rebound of adipose tissue after detraining under the similar inhibition of fat loss. In addition, we partially elucidated the possible mechanism. However, there are several limitations. First, the data of SCAT, VAT mass and serum lipid levels prior to starting training were not analyzed before the start of exercise which might affect the reliability of the present results to some extent. Secondly, the gas metabolism analysis was not performed during the training and detraining in this study and whether the better maintenance of HIIT is related to the higher metabolic rates after detraining needs further investigation. Third, the present study didn’t analyse changes in fat-free mass during training and detraining. HIIT that lasts for > 12 weeks leads to a greater increase in fat-free mass than MICT, resulting in a higher metabolic rate at rest [64]. Therefore, HIIT could be easier to maintain long-term weight loss than MICT. Finally, we did not completely elucidate the related mechanism at the molecular level, including other lipases; fatty acid synthesis; and mechanisms related to the insulin resistance and inflammation.

## Conclusion

HIIT and MICT have a comparable effect on inhibiting fat accumulation in female rats; however, HIIT has a superior effect on inhibiting SCAT and VAT mass increase after short-term training cessation. The present study offers guidance for sustained weight management in women with obesity who discontinue training.

### Electronic supplementary material

Below is the link to the electronic supplementary material.

Supplementary Material 1

Supplementary Material 2

## Data Availability

No datasets were generated or analysed during the current study.

## Abbreviations

HIIT: High-intensity Interval Training
MICT: Moderate-intensity Continuous Training
VAT: Visceral adipose tissue
SCAT: Subcutaneous adipose tissue
ATGL: Adipose Triglyceride Lipase
TG: Triglyceride
TC: Total Cholesterol
LDL-C: Low-density Lipoprotein Cholesterol
HDL-C: High-density Lipoprotein Cholesterol
