# Supplementary material for: The effects of high-intensity interval training/moderate-intensity continuous training on the inhibition of fat accumulation in rats fed a high-fat diet during training and detraining
Source: Lipids Health Dis. 2024 Jul 22;23:221. doi: 10.1186/s12944-024-02209-7 (PMC11265190; doi:10.1186/s12944-024-02209-7)
Supplement: Supplementary file 1 — Supplementary Material 1 [file 12944_2024_2209_MOESM1_ESM.pdf]

1. **High-fat diet(g%):**

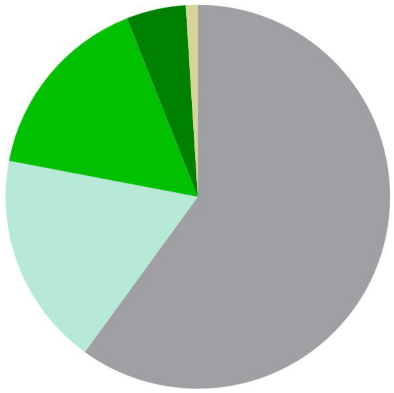

	Protein		Carbohydrate		Fat	
	g(%)	kcal(%)	g(%)	kcal(%)	g(%)	kcal(%)
Chow diet	11.5	9.6	40.4	33.6	2.6	4.8
Egg yolk powder	3.8	3.1	0.2	0.2	13.9	26.0
Sucrose	--	--	16	13.3	--	--
Lard	--	--	--	--	5	9.4
**Total**	**15.3**	**12.7**	**56.6**	**47.1**	**21.4**	**40.2**

2. **Chow diet (Product#: D12450B):**

		g%	kcal%
Protein		19.2	20
Carbohydrate		67.3	70
Fat		4.3	10
	**Total**		100
	**kcal/g**	3.85	
**Ingredient**		**g**	**kcal**
Casein, 80 Mesh		200	800
L-Cystine		3	12
Corn Starch		315	1260
Maltodextrin 10		35	140
Sucrose		350	1400
Cellulose, BW200		50	0
Soybean Oil		25	225
Lard		20	180
Mineral Mix S10026		10	0
DiCalcium Phosphate		13	0
Calcium Carbonate		5.5	0
Potassium Citrate, 1 H2O		16.5	0
Vitamin Mix V10001		10	40
Choline Bitartrate		2	0
FD&C Yellow Dye #5		0.05	0
**Total**		**1055.05**	**4057**
