# Supplementary material for: The effects of high-intensity interval training/moderate-intensity continuous training on the inhibition of fat accumulation in rats fed a high-fat diet during training and detraining
Source: Lipids Health Dis. 2024 Jul 22;23:221. doi: 10.1186/s12944-024-02209-7 (PMC11265190; doi:10.1186/s12944-024-02209-7)
Supplement: Supplementary file 2 — Supplementary Material 2 [file 12944_2024_2209_MOESM2_ESM.pdf]

#1

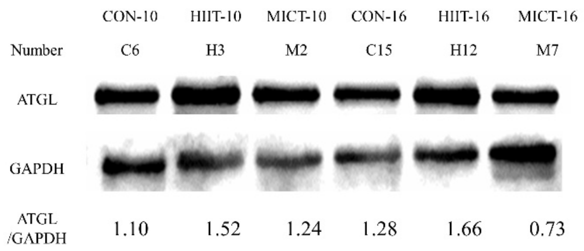
CON-10 HIIT-10 MICT-10 CON-16 HIIT-16 MICT-16
Number C6 H3 M2 C15 H12 M7
ATGL
GAPDH
ATGL /GAPDH 1.10 1.52 1.24 1.28 1.66 0.73

#2

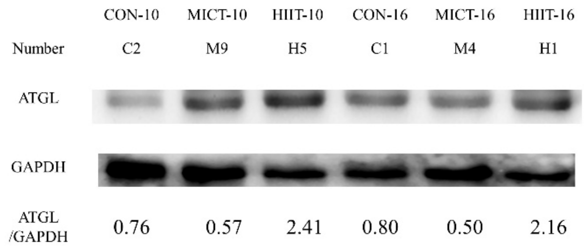
CON-10 MICT-10 HIIT-10 CON-16 MICT-16 HIIT-16
Number C2 M9 H5 C1 M4 H1
ATGL
GAPDH
ATGL /GAPDH 0.76 0.57 2.41 0.80 0.50 2.16

#3

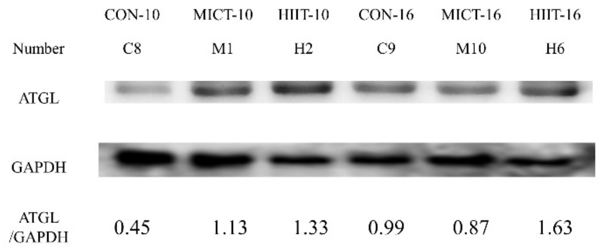
CON-10 MICT-10 HIIT-10 CON-16 MICT-16 HIIT-16
Number C8 M1 H2 C9 M10 H6
ATGL
GAPDH
ATGL /GAPDH 0.45 1.13 1.33 0.99 0.87 1.63
